# Antimicrobial and Anti-Biofilm Peptide Octominin for Controlling Multidrug-Resistant *Acinetobacter baumannii*

**DOI:** 10.3390/ijms22105353

**Published:** 2021-05-19

**Authors:** E. H. T. Thulshan Jayathilaka, Dinusha C. Rajapaksha, Chamilani Nikapitiya, Mahanama De Zoysa, Ilson Whang

**Affiliations:** 1 College of Veterinary Medicine, Chungnam National University, Yuseong-gu, Daejeon 34134, Korea; thimira.thulshan@o.cnu.ac.kr (E.H.T.T.J.); dinusharajapaksha@o.cnu.ac.kr (D.C.R.); chamilani14@cnu.ac.kr (C.N.); 2 National Marine Biodiversity Institute of Korea (MABIK), 75, Jangsan-ro 101 beon-gil, Janghang-eup, Seochun-gun, Chungchungnam-do 33662, Korea

**Keywords:** *Acinetobacter baumannii*, antibacterial, anti-biofilm, bactericidal, Octominin, zebrafish

## Abstract

*Acinetobacter baumannii* is a serious nosocomial pathogen with multiple drug resistance (MDR), the control of which has become challenging due to the currently used antibiotics. Our main objective in this study is to determine the antibacterial and antibiofilm activities of the antimicrobial peptide, Octominin, against MDR *A. baumannii* and derive its possible modes of actions. Octominin showed significant bactericidal effects at a low minimum inhibitory concentration (MIC) and the minimum bactericidal concentration (MBC) of 5 and 10 µg/mL, respectively. Time-kill kinetic analysis and bacterial viability tests revealed that Octominin showed a concentration-dependent antibacterial activity. Field-emission scanning electron microscopy (FE-SEM) analysis revealed that Octominin treatment altered the morphology and membrane structure of *A. baumannii*. Propidium iodide (PI) and reactive oxygen species (ROS) generation assays showed that Octominin increased the membrane permeability and ROS generation in *A. baumannii*, thereby causing bacterial cell death. Further, a lipopolysaccharides (LPS) binding assay showed an Octominin concentration-dependent LPS neutralization ability. Biofilm formation inhibition and eradication assays further revealed that Octominin inhibited biofilm formation and showed a high biofilm eradication activity against *A. baumannii*. Furthermore, up to a concentration of 100 µg/mL, Octominin caused no hemolysis and cell viability changes in mammalian cells. An in vivo study in zebrafish showed that the Octominin-treated group had a significantly higher relative percentage survival (54.1%) than the untreated group (16.6%). Additionally, a reduced bacterial load and fewer alterations in histological analysis confirmed the successful control of *A. baumannii* by Octominin in vivo. Collectively, these data suggest that Octominin exhibits significant antibacterial and antibiofilm activities against the multidrug-resistant *A. baumannii*, and this AMP can be developed further as a potent AMP for the control of antibiotic resistance.

## 1. Introduction

*Acinetobacter baumannii* is a Gram-negative, non-motile, strictly aerobic, non-fermenting coccobacillus and is considered a part of the *Acinetobacter* complex (ABC) consisting of *A. baumannii, A. calcoaceticus,* and genomic species 13TU [1,2]. *A. baumannii* is highly contagious and can cause multiple infections in the human lungs, blood, brain, urinary tract, and skin, leading to multiple complications, such as pneumonia, meningitis, septicemia, urinary tract infections, and abscesses [3]. It can be transmitted via direct contact or contaminated water, food, and even soil. *A. baumannii* infections are commonly seen in patients who have stayed in hospitals for a prolong period of time; hence, it is defined as a nosocomial pathogen [4,5]. Presently, several strains of infectious *A. baumannii* (Ab242, Ab244, and Ab825) have gained resistance to antibiotics (colistin, aminoglycosides, β-lactams, and tetracycline) by developing different mechanisms of action, such as the acquisition of β-lactamases, up-regulation of multidrug efflux pumps, modification of aminoglycosides, permeability defects, and alteration of target sites [6]. Moreover, *A. baumannii* is considered to be a member of ESKAPE (*Enterococcus faecium, Staphylococcus aureus, Klebsiella pneumoniae, A. baumannii, Pseudomonas aeruginosa,* and *Enterobacter spp.*), a high-risk infectious pathogen group mainly responsible for hospital-based infections [7,8]. Accordingly, finding an efficient and commercially available antibiotic has become challenging [9]; hence, there is an urgent need to develop novel therapeutic drug candidates against ESKAPE species.

Currently, a rapid development in the discovery of antimicrobial peptides (AMPs) can be seen, which are used alone or in combination with antibiotics for synergetic and efficient action against microbial infections [10]. AMPs are short-chain polypeptides with less than 50 amino acids and are amphipathic in nature. Based on the secondary structure of AMPs, there are four major types—α-helix, β-sheet, loop, and extended—among which α-helices and β-sheets are prevalent [11]. AMPs have abundant positive amino acids, which result in a net positive charge on the polypeptide chain. Thus, they are easily able to bind to the negatively charged membranes of bacteria, inhibit bacterial growth, and initiate bactericidal actions [12]. Most AMPs are neither limited to a single pathogen nor to a single mode of antimicrobial action, and they show multiple modes of action, such as disruption of membrane and metabolic inhibition of DNA, RNA, protein synthesis, and bacterial wall synthesis [13]. Apart from acting as antimicrobial agents, AMPs show different functions, such as antitumor [14], anti-inflammatory [15], wound-healing [16], and detoxification effects [17]. Hence, it is challenging for pathogens to develop resistance against AMPs [18]. For these reasons, AMPs could be used to solve the global problem of antibiotic-resistant microbial infections.

While various AMPs have been identified from natural sources, problems, such as a low yield and high extraction and purification costs, limit the use of natural AMPs on a large scale [19,20]. Thus, synthetic AMPs present a promising solution to overcome the drawbacks of using natural AMPs. They contain key features based on natural AMPs, with slight modifications to achieve a higher antimicrobial efficiency [21]. Previously, we described the physicochemical and functional properties of Octominin, a newly synthesized AMP derived from the defense protein 3 of *Octopus minor*. Octominin contains a high number of positively charged residues (17% R and 17% H), with a net charge of +5 and hydrophobic residues (17% I, 8% L, 13% A, and 4% W) and a total hydrophobic ratio of 43%. The protein-binding potential (Boman index) of Octominin is 1.86 kcal/mol, and its secondary structure is an α-helix. Moreover, Octominin shows a potent anti-Candidal activity against *Candida albicans* [22].

In this study, we tested Octominin against multidrug-resistant (MDR) *A. baumannii* infection in vitro to determine its antibacterial activity. Initially, the antibiotic resistance of *A. baumannii* was assessed. To evaluate the antibacterial activity of Octominin, the minimum inhibitory concentration (MIC) and minimum bactericidal concentration (MBC) were determined. Furthermore, we tested alterations in the morphology and ultrastructure, membrane permeability, reactive oxygen species (ROS) generation, lipopolysaccharide (LPS) neutralization, and biofilm inhibition and eradiation activities to determine its mode of action against *A. baumannii.* We evaluated its toxicity using hemolysis and cell viability assays in mammalian cells. Additionally, the in vivo efficacy of Octominin was tested using an *A. baumannii*-infected zebrafish model. Finally, based on our findings, we suggest that the novel AMP Octominin discovered in this study can be used as a promising solution for the control of the antibiotic-resistant *A. baumannii.*

## 2. Results

### 2.1. Isolation and Molecular Identification of A. baumannii

For the identification of the bacteria, 16s rRNA sequencing was performed, and the sequence was blasted in the GenBank of National Center for Biotechnology Information (NCBI). The nucleotide BLAST search results identified our isolate up to the species level. It had a 99.87% similarity with the *A. baumannii* strain, L30; accession no. KU922292. Thus, the identified bacteria were named *A. baumannii* (Lab strain 1).

### 2.2. Antibiotic Sensitivity Spectrum of A. baumannii

The antibiotic disc diffusion method was used to determine the antibiotic sensitivity of *A. baumannii*. Among the 18 antibiotics tested, the majority (55.5%) (gentamycin, streptomycin, vancomycin, cefotaxime, penicillin, erythromycin, clindamycin, tobramycin, trimethoprim, and rifampin) were resistant to *A. baumannii.* Of these, vancomycin, penicillin, erythromycin, clindamycin, trimethoprim, and rifampin showed zero zones of inhibition. Moreover, tetracycline, amikacin, ciprofloxacin, and sulfamethoxazole/trimethoprim exhibited an intermediate resistance (22.2%). Only four antibiotics (chloramphenicol, ofloxacin, doxycycline, and imipenem) were susceptible (22.2%) and showed a complete growth inhibition of *A. baumannii* (Table 1).

The zones of inhibition were measured around the 6 mm Sensi-Discs (Dickinson and Company, Sparks, MD, USA) and compared with the BD BBL^TM^ Zone Interpretation Chart (Dickinson and Company, Sparks, MD, USA). The strains were then classified as resistant, intermediate, or susceptible.

### 2.3. MIC and MBC of A. baumannii Treated with Octominin

Broth microdilution and agar plating methods were used to determine the MIC and MBC of Octominin against *A. baumannii,* respectively. Octominin showed a strong antibacterial activity, with an MIC and MBC of 5 and 10 µg/mL, respectively, representing an MBC/MIC ratio of 2.0. The commercial antibiotic chloramphenicol (positive control) also showed antibacterial activity, with MIC and MBC values of 10 and 50 µg/mL, respectively. 

### 2.4. Time-Kill Kinetics and Bacterial Viability of A. baumannii upon Octominin Treatment

**Figure 1:**
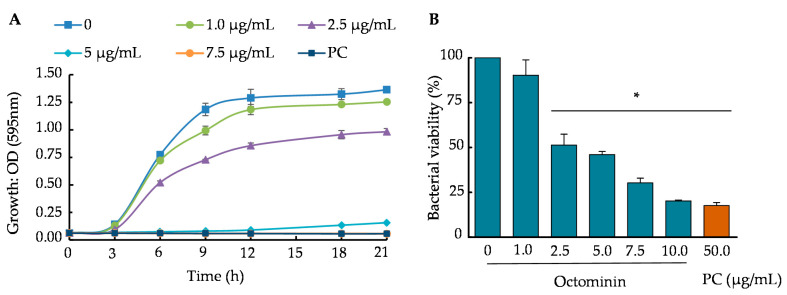
Time-kill kinetic assay and bacterial viability of *A. baumannii* upon treatment with Octominin. (**A**) The time-kill kinetics of *A. baumannii* treated with Octominin (1.0, 2.5, 5.0, and 7.5 µg/mL) were assessed at every 3 h intervals by measuring the optical density at 595 nm (OD_595_). Phosphate-buffered saline (PBS) and 50 µg/mL chloramphenicol were used as the negative and positive controls, respectively. The bars indicate the mean ± standard deviation (*n* = 3). (**B**) *A. baumannii* viability upon Octominin treatment. *A. baumannii* was treated with Octominin at increasing concentrations (0–10 µg/mL) and positive-control chloramphenicol. The samples were incubated for 24 h. Later, a 3-(4,5-dimethylthiazol-2-yl)-2,5-diphenyltetrazolium bromide (MTT) assay was performed, and the OD_595_ was measured. * *p* < 0.05 compared to the control (0) group. The error bars indicate the mean ± standard deviation (*n* = 3). PC = Positive control (chloramphenicol).

Time-kill kinetic and 3-(4,5-dimethylthiazol-2-yl)-2,5-diphenyltetrazolium bromide (MTT) assays were conducted to determine the growth and viability reduction in Octominin-treated *A. baumannii*. Octominin inhibited bacterial growth in a concentration-and time-dependent manner in the time-kill kinetic assay (Figure 1A). In the negative control and Octominin concentrations below the MIC, *A. baumannii* grew with a pattern of a sigmoid curve (typical growth curve) until 21 h, showing the highest end optical density (OD_595_) value in the control, followed by gradually lowered end point OD_595_ values for the respective increased concentrations. At MIC, a clear inhibition was observed up to 12 h, and thereafter, a slight reduction of inhibition was observed. When the concentration of Octominin was higher (7.5 µg/mL) than the MIC, the bacterial growth was minimized, showing a growth inhibition similar to that with chloramphenicol (50 µg/mL). The bacterial viability percentage changed in a concentration-dependent manner with Octominin (0–10 µg/mL) (Figure 1B). The highest viability was observed in the negative control (100%). At the MIC and MBC of Octominin, the *A. baumannii* viability was significantly decreased (*p* < 0.05) to 46% and 20.28%, respectively. Moreover, these values were almost the same (17.6%) as those obtained after treatment with chloramphenicol (50 µg/mL).

### 2.5. Morphological Changes in A. baumannii Treated with Octominin

**Figure 2:**
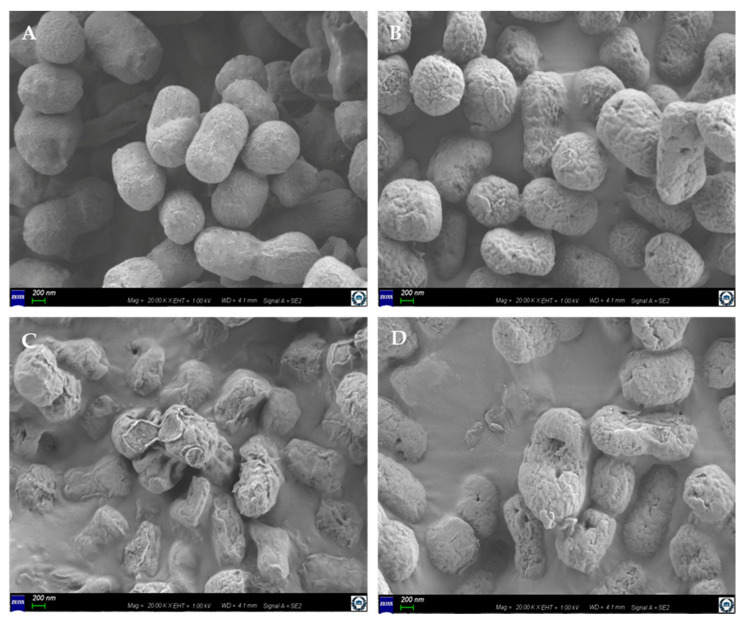
Effect of Octominin on the morphological and structural changes in *A. baumannii*. Bacteria, which were (**A**) untreated, (**B**) treated at MIC (5 μg/mL) or (**C**) at MBC (10 μg/mL) of Octominin, or (**D**) treated with chloramphenicol (50 μg/mL), assessed under a field-emission scanning electron microscope (FE-SEM). Scale bar represents 200 nm.

Field-emission scanning electron microscopy (FE-SEM) analysis revealed that *A. baumannii* treated with the MIC (Figure 2B) and MBC (Figure 2C) of Octominin showed distinguishable morphological changes, compared to the phosphate-buffered saline (PBS)-treated negative control (Figure 2A). Undamaged smooth cell surfaces were observed in the negative control, while the *A. baumannii* cells treated with the MIC of Octominin had rough bacterial cell surfaces, with structural alterations and damage to the cell membrane and small holes. Moreover, the sample treated at MBC showed greater morphological and structural changes, including cell shrinkage, cell membrane damage, and a higher number of pores, than the MIC-treated sample. Interestingly, the damage was more potent at the MBC of the Octominin-treated group than that of the positive control, which was treated with chloramphenicol (Figure 2D).

### 2.6. Octominin Effects on A. baumannii Membrane Permeability and Cell Death

**Figure 3:**
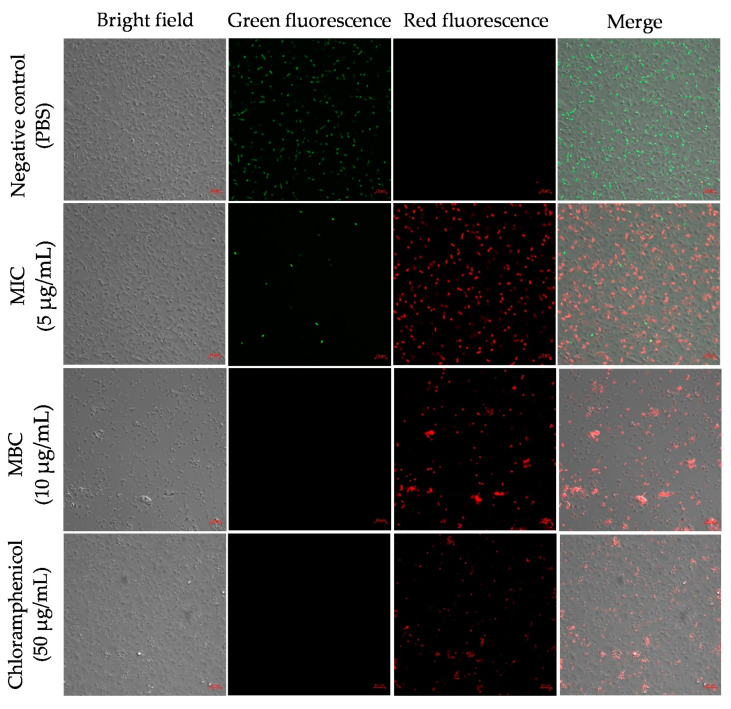
Effect of Octominin on membrane permeability alteration in *A. baumannii*. PBS-treated *A. baumannii*, Octominin-treated *A. baumannii* at MIC (5 μg/mL) and MBC (10 μg/mL), and chloramphenicol-treated *A. baumannii* were incubated for 10 h and stained with Propidium iodide (PI) and Fluorescein diacetate (FDA). The cells were visualized through confocal microscopy (CFMC) for red fluorescence (PI) and green fluorescence (FDA) at excitation and emission wavelengths of 535 nm and 617 nm, respectively, and 488 nm and 535 nm, respectively. Scale bar represent 5 µm.

We conducted propidium iodide (PI) and fluorescein diacetate (FDA) staining to determine the membrane permeability and detect live cells in *A. baumannii* after treatment with Octominin at MIC and MBC (Figure 3). PI can only enter the permeability-altered cells and bind with their nuclei to produce red fluorescence. FDA produces green fluorescence in viable cells. In this study, the negative control treated with PBS showed almost all cells with green fluorescence and no red fluorescence, indicating that all bacterial cells were alive. Samples of Octominin treated at MIC exhibited mainly red fluorescence, as opposed to green fluorescence, demonstrating that the majority of the cells underwent cell permeability changes. Moreover, the MBC-treated sample exhibited red fluorescence in all cells, with the absence of green fluorescence. Furthermore, the MBC-treated sample had a lower number of cells than the MIC-treated sample. The positive control showed a similar pattern of red staining to that of the sample that was treated with Octominin at MBC.

### 2.7. ROS Generation in A. baumannii upon Exposure to Octominin

**Figure 4:**
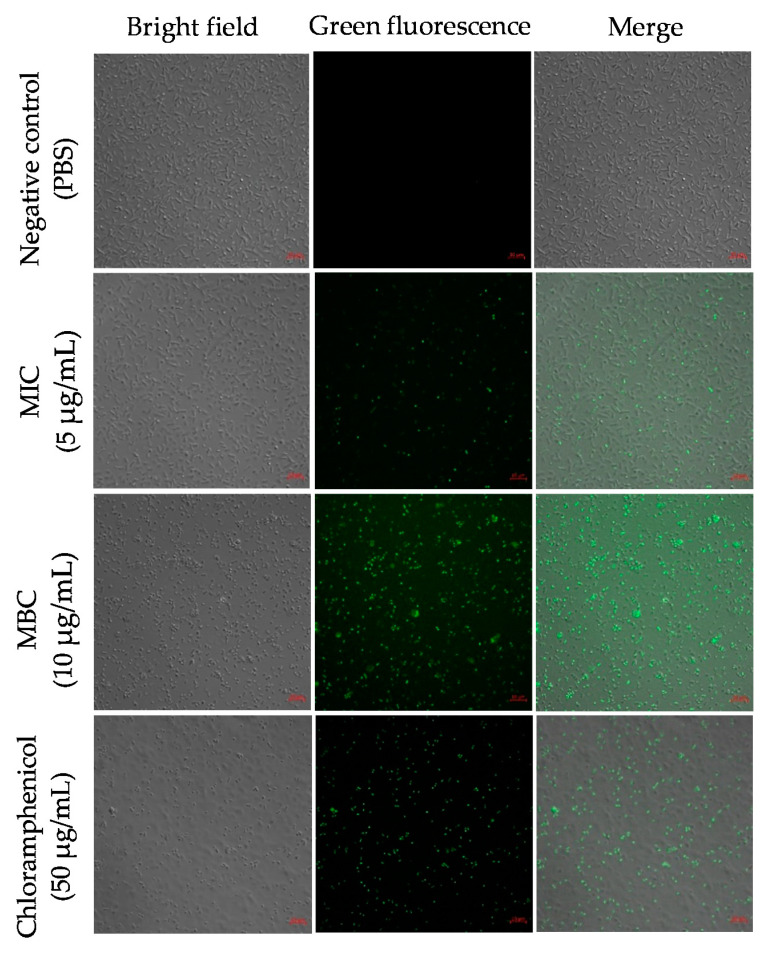
Effect of Octominin on the reactive oxygen species (ROS) generation in *A. baumannii*. PBS-treated *A. baumannii*, Octominin-treated *A. baumannii* at MIC (5 μg/mL) and MBC (10 μg/mL), and chloramphenicol-treated *A. baumannii* were incubated for 10 h and stained with 2′,7′-Dichlorodihydrofluorescein diacetate (H_2_DCFDA). The cells were visualized through confocal microscopy (CFMC) for green fluorescence at excitation and emission wavelengths of 488 nm and 535 nm, respectively. Scale bar represents 5 µm.

To determine the ROS levels upon Octominin treatment in *A. baumannii*, we performed 5-(and-6)-carboxy-2′,7′-dichlorodihydrofluorescein diacetate (H_2_DCFDA) staining. The cells with a higher ROS production were detected by green fluorescence upon staining with H_2_DCFDA. The negative control showed no fluorescence, with no generation of ROS (Figure 4). Samples treated at the MIC of Octominin showed a lower amount of green fluorescence than those treated at MBC, indicating Octominin concentration-dependent ROS production. In the positive control, a similar pattern of green fluorescence was observed to that in the samples treated at MBC.

### 2.8. LPS Neutralization Activity of Octominin

**Figure 5:**
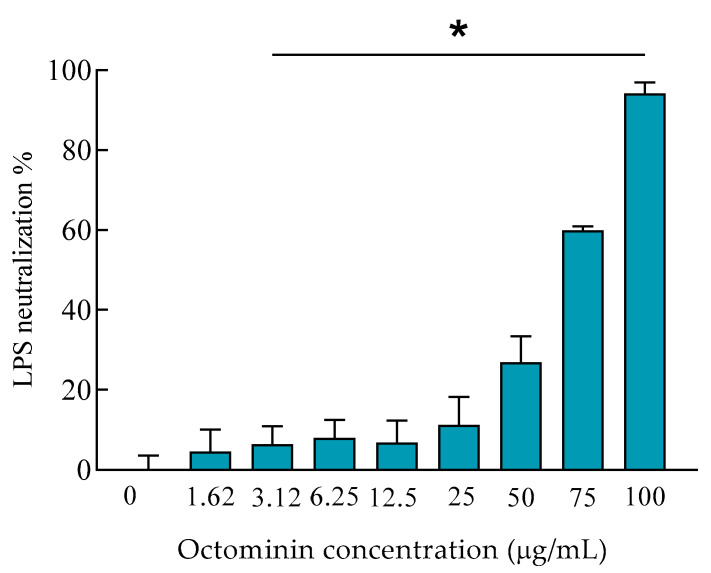
Lipopolysaccharide (LPS) neutralization activity of Octominin. LPS (0.5 EU/mL) was reacted with Octominin (0–100 µg/mL) and incubated for 30 min at 37 °C. The remaining LPS after the neutralization was quantified by the LAL chromogenic endotoxin quantitation kit, with an absorption measurement of colored substrate at 410 nm. * *p* < 0.05, compared to the negative control (0-Octominin) group. The error bars indicate the mean ± standard deviation (*n* = 3).

We conducted a limulus amebocyte lysate (LAL) test to assess the LPS neutralization activity of Octominin. Initially, 0.5 EU/mL LPS was reacted with different concentrations of Octominin and remained LPS after the neutralization was quantified by LAL in the presence of chromogenic substrate. As shown in Figure 5, direct LPS neutralization activity was observed with an Octominin concentration-dependent manner. Nonetheless, low-concentration Octominin samples did not produce a high neutralization percentage, and when the Octominin concentration increased, a gradual and higher percentage of LPS neutralization was observed than the untreated sample. Moreover, significantly (*p* < 0.05) high LPS neutralization (94.24%) was seen at the highest tested concentration (100 µg/mL) of Octominin.

### 2.9. Biofilm Inhibition and Biofilm Eradication Activities of Octominin

**Figure 6:**
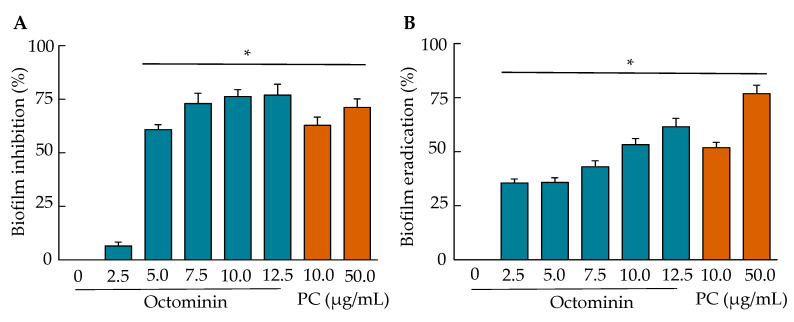
Anti-biofilm effect of Octominin against *A. baumannii*. (**A**) Quantitative measurement of the biofilm formation inhibition by Octominin. *A. baumannii* was treated with increasing concentrations of Octominin (0–12.5 µg/mL) and chloramphenicol (10 µg/mL and 50 µg/mL). After 24 h of treatment, the remaining biofilm was stained with crystal violet (CV), and the OD was measured at 595 nm (OD_595_); (**B**) Quantitative measurement of *A. baumannii* biofilm eradiation by Octominin. *A. baumannii* was allowed to form biofilm for 24 h and then the biofilm was treated with increasing concentrations of Octominin (0–12.5 µg/mL) and chloramphenicol (10 µg/mL and 50 µg/mL). After 24 h of treatment, the remaining biofilm was stained with CV, and the OD_595_ was measured. * *p* < 0.05, compared to the control (0-Octominin) group. The error bar indicates the mean ± standard deviation (*n* = 3). PC = Positive control (chloramphenicol).

The biofilm formation inhibition and eradication upon Octominin treatment were quantified using crystal violet (CV) staining, as shown in Figure 6. The lowest biofilm inhibition was detected at 2.5 µg/mL of Octominin, and then an Octominin concentration-dependent inhibition was observed (Figure 6A). At the MIC (5 µg/mL) and MBC (10 µg/mL) of Octominin, the inhibition was significantly increased (*p* < 0.05) by 61.59% and 76.29%, respectively, compared to that in the negative control. Comparatively, at MBC (50 µg/mL), chloramphenicol showed a lower inhibition (71.45%) than Octominin MBC. Similar to the biofilm inhibition results, the biofilm eradication results also demonstrated a concentration-dependent biofilm eradication effect of Octominin (Figure 6B). The results showed that preformed biofilms could be eradicated significantly (*p* < 0.05) by 35.62% and 53.19% at the MIC (5 µg/mL) and MBC (10 µg/mL) levels of Octominin, respectively; contrastingly, the MBC (50 µg/mL) of chloramphenicol caused a significantly (*p* < 0.05) high eradication (76.19%) of preformed biofilms, compared to that of the negative control. 

### 2.10. Hemolysis Activity and Cytotoxicity of Octominin

**Figure 7:**
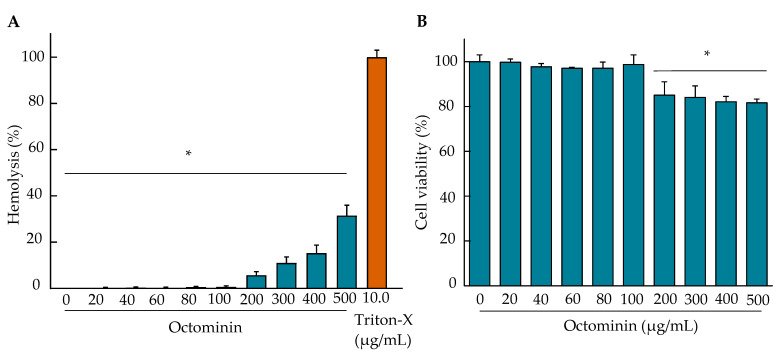
Hemolytic activity and cytotoxicity of Octominin. (**A**) Mouse red blood cells (RBC) were treated with Octominin (0–500 µg/mL) for the determination of the hemolytic activity. Nuclease-free water and Triton-X (10 µg/mL) were used as the negative and positive controls, respectively. The samples were incubated for 1 h, and the RBCs were removed by centrifugation. The absorbance of the supernatant was measured at 414 nm. * *p* < 0.05, compared to the positive control (Triton-X, 10 µg/mL) group. The error bars indicate the mean ± standard deviation (*n* = 3). (**B**) Cytotoxicity of Octominin against murine Raw 264.7 macrophage cells using an MTT assay. The cells (2.0 × 10^5^ cells/mL) were seeded onto 96-well plates (100 µL/well) and incubated for 12 h. Octominin was added to each well at 0–500 µg/mL, and the plate was incubated at 37 °C for 24 h in a 5% CO_2_ incubator. The supernatant was replaced with fresh media and treated with the MTT reagent and incubated for 4 h. Then, dimethyl sulfoxide (DMSO) (50 µL) was added and incubated for 30 min with constant shaking on a plate shaker. The absorbance was measured at 595 nm. * *p* < 0.05, compared to the negative control (0-Octominin) group. The error bars indicate the mean ± standard deviation (*n* = 3).

Hemolysis and cell viability assays were performed to determine the toxicity of Octominin in mammalian cells (Raw 264.7 macrophage). As shown in Figure 7, up to a concentration of 100 µg/mL, Octominin showed almost zero hemolysis, which is similar to the negative control. However, an increased hemolysis was observed when the concentration of Octominin increased. The maximum hemolysis was 31.50% at the tested maximum concentration of 500 µg/mL, which was significantly (*p* < 0.05) lower than that of the positive control (100%). Figure 6B shows the cytotoxicity of Octominin in RAW 264.7 cells. Different concentrations of Octominin-treated RAW 264.7 macrophages showed no significant (*p* > 0.05) cell viability changes up to 100 µg/mL, compared to that in the negative control. At 200 µg/mL and higher Octominin concentrations, the cells showed a significant (*p* < 0.05) reduction in cell viability, ranging from 86.53–80.70%, compared to that in the negative control. Apart from the cell viability, no morphological changes were observed in the cells at the tested concentrations.

### 2.11. Efficiency of A. baumannii Control by Octominin in a Zebrafish Model

**Figure 8:**
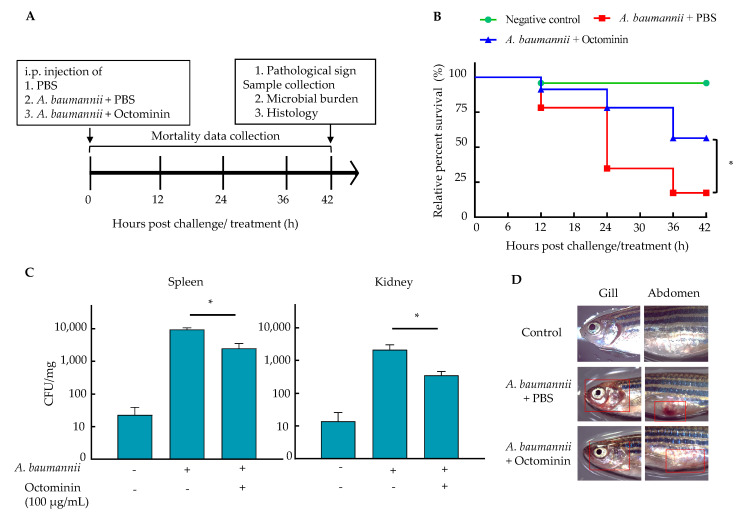
In vivo analysis of Octominin in *A. baumannii*-challenged adult zebrafish. (**A**) Schematic diagram of the experimental process. Adult zebrafish were challenged with 20 µL of *A. baumannii* culture (2.1 × 10^11^ CFU/mL) intraperitonially (i.p.) and simultaneously treated with 10 µL of Octominin (10 mg/mL) and PBS as the control. Uninfected fish were maintained as the negative control. The fish were maintained at 28 °C. (**B**) RPS after treatment over 42 h. The rate of surviving zebrafish in each group is depicted (*n* = 24/group). (**C**) The microbial count in the spleen and kidney with *A. baumannii* infection. The spleen and kidney were isolated after 42 hpi, homogenized in PBS, plated on trypticase soy agar (TSA), and incubated. The CFU were counted, and the average CFU/mg was derived. (**D**) Pathological clinical signs of *A. baumannii* infection in zebrafish. The abdomen and gill area of the zebrafish (marked in red frames) were observed under a light microscope to detect clinical signs of infection. * *p* < 0.05, compared to the PBS-treated negative control group. The error bars indicate the mean ± standard deviation (*n* = 3).

Zebrafish were intraperitoneally (i.p.) infected with *A. baumannii* and treated with Octominin to study the effectiveness of Octominin in vivo. Based on the preliminary data, 20 µL of the 2.1 × 10^11^ CFU/mL concentration of *A. baumannii*, which produced 80% mortality in zebrafish, was selected as the optimum bacterial concentration for the in vivo study. As shown in Figure 8B, the survival data of the in vivo study revealed that the Octominin 100 µg/fish-treated group had a 54.1% relative percentage survival (RPS) at the end of the study, which is significantly higher than that of the PBS-treated group (16.6%) (*p* < 0.05). Meanwhile, the uninfected group had 95.8% RPS at 42 h post challenge/ treatment. The microbial burden data revealed significantly reduced (*p* < 0.05) colony-forming units (CFUs) in both the spleen and kidney in the Octominin-treated group, compared to those in the PBS-treated group (Figure 8C). The Octominin-treated group had an average of 2496 CFU/mg and 357 CFU/mg in the spleen and kidney, respectively, while the PBS-treated *A. baumannii* group had 9450 CFU/mg and 2143 CFU/mL in the spleen and kidney, respectively.

**Figure 9:**
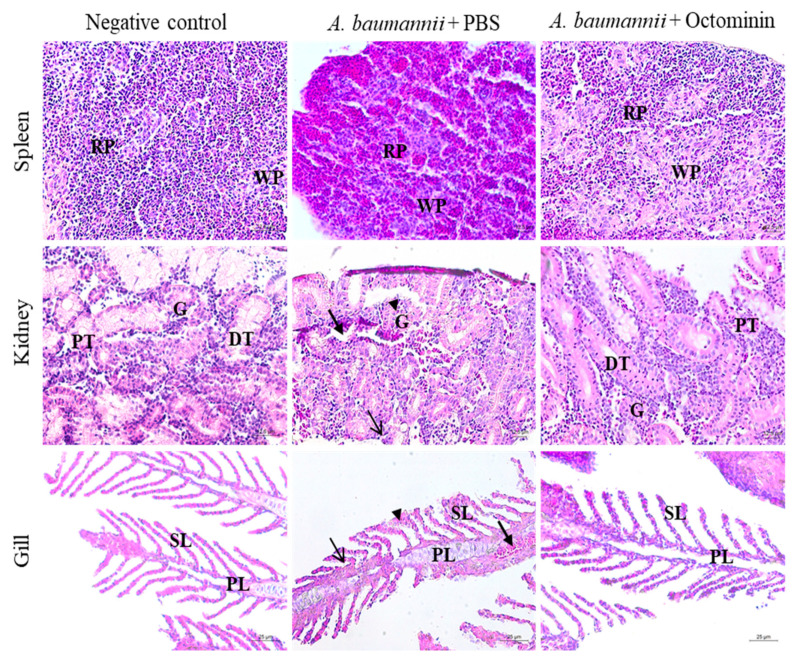
Histological analysis of zebrafish spleen, kidney, and gill in the negative control, *A. baumannii*-infected, and PBS-treated, *A. baumannii*-infected, and Octominin-treated groups. The uninfected groups showed no deviation from the normal structure in all three tissue samples. However, *A. baumannii*-infected zebrafish spleen had a relatively higher amount of red pulp (RP) than white pulp (WP), while the Octominin-treated sample showed a regular arrangement of the spleen tissue. Kidney samples of *A. baumannii*-infected and PBS-treated zebrafish had shrunken glomeruli (G), with an increased Bowman space (arrowhead), macrophage infiltration (thin arrow), and cellular necrosis (thick arrow). However, the Octominin-treated group showed fewer deviations, with regular glomeruli, proximal tubule (PT), distal tubule (DT) and a few macrophages. The gill tissue showed erythrocyte infiltration (thick arrow), thickening in primary lamella (PL) (thin arrow), and secondary lamella (SL) clubbing (arrowhead) in *A. baumannii*-infected and PBS-treated zebrafish, while the Octominin-treated zebrafish showed comparatively fewer deviations. Scale bar represents 12.5 µm in spleen and kidney tissues and 25 µm in gill tissues.

Pathological clinical signs revealing possible internal hemorrhages caused by *A*. *baumannii* infection included red colored gills and abdomen area in zebrafish of the PBS-treated *A. baumannii* group. However, these signs were present to a lesser extent in the Octominin-treated *A. baumannii* group (Figure 8D). Histological analysis also supported the pathological clinical signs of zebrafish in reducing the inflammatory conditions that were caused in the gill, spleen, and kidney (Figure 9). In particular, the spleen showed lymphocyte infiltration and a higher amount of red pulp than white pulp in the PBS-treated sample. In contrast, a regular density of white and red pulp was observed in the Octominin-treated group. In zebrafish kidneys infected with *A. baumannii,* shrunken glomeruli, an increased Bowman space, macrophage infiltration, and cellular necrosis with a low cell density were observed. However, Octominin-treated zebrafish kidneys showed comparatively less damage, with undamaged glomeruli and the absence of macrophage infiltration. Furthermore, histology of the gill revealed significant damage to the primary lamella, with erythrocyte infiltration and clubbing of the secondary lamella in the PBS-treated group, while no significant damage was observed in the Octominin-treated zebrafish gill.

## 3. Discussion

AMPs are one of the important immune molecules. They form the first line of defense in innate immunity, which protects the host against invading pathogens. Due to their rapid killing effect, wide spectrum of activities, broad mechanisms of action, and low possibility of developing resistance, AMPs (natural and synthetic) are considered as next-generation antimicrobials for controlling MDR microbes [23]. Due to the rising tendency of microbes to develop resistance to commercially available antibiotics, much attention has been paid to the search for novel treatment strategies against the MDR and hospital-derived infectious *A. baumannii* [24].

In this study, we tested the antibiotic resistance level of *A. baumannii* (Lab strain 1) and investigated the antibacterial and antibiofilm activities of the AMP, Octominin. Finally, the effectiveness of the in vivo control of *A. baumannii* was evaluated using a zebrafish model. The tested *A. baumannii* strains were resistant to the main antibiotic classes, such as aminoglycosides, glycopeptides, macrolides, cephalosporins, penicillin, lincosamide, diaminopyrimidine, and rifamycin. Only 22.2% of the tested antibiotics were susceptible. Similar to our findings, Son et al. [25] isolated an *A. baumannii* strain from a patient with community-acquired pneumonia and found the same pattern of antibiotic resistance for 10 major antibiotics, showing sensitivity to four antibiotics (colistin, gentamicin, minocycline, and tigecycline). The MDR of *A. baumannii* is mainly due to the involvement of antibiotic resistance mechanisms, such as antibiotic-inactivating enzymes (β-lactamases, oxcillinases, and carbapenemases), active efflux pumps, and bacterial natural resistance due to poor membrane penetration [26]. Our findings suggest that the newly isolated *A. baumannii* (Lab strain 1) already consists of multiple antibiotic resistance due to the development of one or more antimicrobial resistance factors. This further reinstates the urgency of the search for new antibacterial agents to control MDR *A. baumannii* in addressing the global public health threat of the emergence of drug-resistant bacteria.

Previously, we studied the antifungal and anti-inflammatory antibacterial activities of the 23-amino acid length of the synthetic peptide, Octominin [22,27]. Since the amino acid sequence of Octominin contained the basic features of antibacterial peptides, we aimed to determine whether it has antibacterial activity against *A. baumannii.* Our results showed that Octominin exhibited growth inhibition and bactericidal activities against *A. baumannii* at low concentrations (MIC, 5 µg/mL; MBC, 10 µg/mL), with an MBC/MIC ratio of 2.0. The chloramphenicol-treated groups had two- and five-times higher MIC (10 µg/mL) and MBC (50 µg/mL) values than the Octominin-treated groups, respectively, with a high MBC/MIC ratio of 5.0. Previously, several AMPs, such as LL-37, a human cathelicidin AMP (MIC, 32 μg/mL; MBC, 128 μg/mL; MBC/MIC, 4) [28], and omiganan, a synthetic AMP (MIC, 32 μg/mL; MBC, 64 μg/mL; MBC/MIC, 2) [29] with antibacterial activities, have been tested against *A. baumannii*. In this study, lower MIC and MBC values suggest that Octominin is a comparatively effective antibacterial agent and is more efficient than the conventional antibiotic, chloramphenicol. Moreover, according to the criteria described by Traczewski et al. [30], the low MBC/MIC ratio indicates that Octominin has a high bactericidal activity, rather than bacteriostatic activity. The time-kill kinetic and bacterial viability assays confirmed the concentration-dependent bactericidal action of Octominin against *A. baumannii.* This could be associated with the physicochemical properties of Octominin (short chain, α-helix, net positively charge (+5), amphipathic nature) that enable it to interact with negatively charged molecules on the surface of *A. baumannii* and penetrate the membrane efficiently, which is related to the mechanism of bactericidal action [31]. Collectively, our data indicate that apart from the anticandidal activity (MIC, 50 μg/mL; minimum fungicidal concentration, 200 μg/mL) [22], Octominin also exhibits a potent antibacterial activity against *A. baumannii*.

Previously, a number of AMPs have been tested for *A. baumannii*, and different modes of action have been described. For instance, LL-37 shows membrane disruption [32], Mastoparan acts as a cell membrane-penetrating peptide [33], and ZY4 causes membrane damage, pore formation, and permeabilization of the bacterial membrane [23]. Moreover, AMP Ω76, which was produced by the maximum common subgraph approach, is also known to produce ultrastructural changes in some sensitive *A. baumannii* strains [34]. In this study, Octominin caused significant structural alterations on the surface of *A. baumannii* cells, and the severity of the morphological changes, shrinkage, and pore-like damage was concentration-dependent and more potent than that observed with chloramphenicol. This clearly demonstrates that Octominin damaged the *A. baumannii* membrane. This was also supported by PI influx, which showed that Octominin was able to permeabilize the bacterial membrane in a concentration-dependent manner. Therefore, we propose that Octominin permeabilizes and damages the bacterial membrane of *A. baumannii*, resulting in an efficient bactericidal activity. Moreover, our results indicate that an increased *A. baumannii* membrane permeability might not be the sole cause of cell death; however, it might lead to the “self-promoted” uptake of Octominin. It also suggests that Octominin might have other intracellular targets or other modes of action against *A. baumannii*, as described for the anticandidal activity against *A. albicans* [22], which needs to be elucidated in more detail.

The ROS induction ability of AMPs has been well documented. When bacteria are exposed to antimicrobial agents, ROS are generated inside bacterial cells due to cellular stress, causing further damage to bacteria and initiating bactericidal action [35]. For example, HD5, a human-based cationic polypeptide, shows antimicrobial activity against *A. baumannii* by reducing the activity of superoxide dismutase and catalase, ultimately increasing the ROS in bacteria [36]. In this study, ROS-mediated killing was demonstrated by H_2_DCFDA staining in *A. baumannii* cells, following Octominin treatment at MIC and MBC, compared to the negative control. ROS can affect a range of molecules and pathways, including proteins (by oxidation) and lipids (by peroxidation), and also lead to nucleotide damage, enzyme inhibition, and the activation of programmed cell death [37]. Thus, it is difficult to determine which event leads to the loss of viability of bacterial cells following damage. However, considering all the results, as reported previously, we confirmed that Octominin has a multimodal action. Moreover, due to the multiple targets of action (morphological changes, membrane damage and permeability, and ROS formation), the propensity for the rapid development of resistance due to the prolonged use of the peptide (Octominin) against *A. baumannii* may be minimal. 

LPS is considered as a major endotoxin in Gram negative bacteria, and it activates inflammatory pathways through the induction of a distinctive pattern of cytokine release [38]. The neutralization of LPS is the most efficient method for the control of inflammatory conditions in bacterial infections. Various AMPs have been tested for their LPS neutralization activity, and they can block LPS-mediated inflammations through the recognition and binding of circulating LPS molecules, disturbing the local membrane environment of the receptors (TLR4) by modifying its activation state and inhibition of proinflammatory genes for LPS [39]. Unno et al. found that *A. baumannii*-derived LPS can function as a signaling molecule that impacts the inflammatory activity of white adipose tissue [40]. Similar to the antimicrobial activity of Octominin against *A. baumannii*, it showed a significant concentration-dependent LPS neutralization capability in the LAL test. However, the precise mode of action must be derived for the Octominin in the neutralization activity of LPS in future.

One of the challenges associated with antibiotic-resistant bacteria, including *A. baumannii*, is the formation of biofilms. Biofilms allow the bacteria to adhere to each other and attach to the surfaces.They consist of complexes of single or multiple types of microorganisms with extracellular polymeric substances (exopolysaccharides, proteins, lipids, and DNA) [41]. Due to the lower antibiotic penetration capacity through biofilms, antibiotic trapping, antibiotic-degrading enzyme activities in biofilms, and low metabolic state at the basement layer of the biofilm, controlling the biofilm-forming bacteria is crucial [42,43]. Considering an AMP as an antibiofilm agent, it should reduce the formation of biofilms by inhibiting the bacterial growth in the initial stage and eradicate the mature biofilm that has already been formed [44]. Peng et al. [45] demonstrated that the peptide, Cec4, has remarkable biofilm formation inhibition and eradication activities against clinical isolates of *A. baumannii*. They also showed that a biofilm formation of 50% or higher could be reduced with a low dose of this peptide. Consistent with these findings, Octominin was able to inhibit *A. baumannii* biofilm formation by more than 75% at the MBC (10 µg/mL) level, and it was higher than that when 50 µg/mL chloramphenicol was used. This suggests that Octominin acts as an antibiofilm agent probably by exerting its bactericidal action, ultimately disturbing the formation of the *A. baumannii* biofilm. Moreover, we showed that Octominin was able to eradicate established biofilms by 53.19% at the MBC (10 µg/mL) level, which was similar to the chloramphenicol activity at the same concentration. These results suggest that Octominin has a strong antibiofilm activity against MDR *A. baumannii* isolates, which may be due to the penetration of Octominin via the biofilm matrix. However, we noticed that the antibiofilm activity of Octominin was relatively lower (about 50% of the remaining biofilm at 10 µg/mL) on the preformed biofilms than the biofilm formation inhibition, because the disruption of preformed biofilm is more difficult than biofilm inhibition [46]. Consistent with these results, Kim et al. also showed that a significant amount of biofilm remained after the biofilm eradication assay on *A. baumannii* with magainin 2 [31]. Collectively, our data indicate that Octominin is proficient in inhibiting biofilm formation and disrupting preformed biofilms effectively and could be further used as a promising candidate for the development of antimicrobial agents against MDR *A. baumannii.*

When using AMPs as therapeutic agents, toxicity to mammalian cells is one of the major limitations for the further development of drug candidates for clinical trials or commercial use in humans [47]. Hemolysis and lymphocyte cell viability assays are the two main tests used to detect AMP toxicity [48]. In this study, Octominin at MIC (5 µg/mL) and MBC (10 µg/mL) showed almost 0% hemolysis. Even at high concentrations (500 µg/mL), it had a low hemolytic activity (31.50%). Moreover, no significant reduction in cell viability was observed in RAW 264.7 cells up to 100 µg/mL, which is in accordance with the previously reported results, wherein up to 100 µg/mL of Octominin was reported to be safe to use in human embryonic kidney 293 (HEK293) cells, and it produced cytoprotective activity in LPS induced RAW 264.7 cells without any cytotoxicity even at high concentrations such as 500 µg/mL [22,23]. Since Octominin had a low MBC (10 µg/mL) against *A. baumannii*, we used Octominin for the in vivo control of *A. baumannii* using a zebrafish model.

While *A. baumannii* infection has been well established in murine models, there is very limited data available on *A. baumannii* infection in a fish model [49]. For the first time, we were able to establish an *A. baumannii* infection in zebrafish and test its effectiveness. At a high density (2.1 × 10^11^ CFU/mL) of *A. baumannii*, excessive mortality was observed in zebrafish (85.4%). However, with Octominin treatment, *A. baumannii*-infected zebrafish had a significantly high (*p* > 0.05) RPS of 54.7%. Furthermore, a significantly low bacterial burden was observed in the kidney and spleen tissues in the Octominin treatment group, which also confirms the successful in vivo bactericidal activity of Octominin against *A. baumannii.* Furthermore, we compared the pathological indications of the fish with histological analysis to understand the tissue-level damage caused by *A. baumannii* and the effect of Octominin in controlling this damage and alterations. Notably, the observed red coloration in the gill area and abdomen indicated possible inflammatory conditions, which was confirmed by histological analysis. In *Aeromonas hydrophila,* infection in channel catfish (*Ictalurus punctatus*), glorumuli shrinkage with an expanded Bowman space, cellular necrosis, and macrophage infiltration were observed in the kidney, while gills exhibited clubbing of the lamella and erythrocyte infiltration [50]. A similar pattern of tissue damage was observed in *A. baumannii*-challenged zebrafish gill and kidney; however, less damage was observed in the spleen, with a high density of red pulp. However, these damages were remarkably reduced in the Octominin-treated group, demonstrating the effectiveness of the control of *A. baumannii* in vivo.

## 4. Materials and Methods

### 4.1. A. baumannii (Lab Strain 1), Media, and Growth Conditions

The *A. baumannii* (Lab strain 1) used in this study was isolated from natural stream water in the Korea and confirmed by 16S rRNA sequencing (Cosmogenetech, Seoul, Korea) using universal primers, 27F-forward primer (5′-AGAGTTTGATCMTGGCTCAG-3′) and 1492R-reverse primer (5′-TACGGYTACCTTGTTACGACTT-3′). For each test, *A. baumannii* were used at a bacterial cell density of 1 × 10^6^ CFU/mL (OD_595_–0.08), unless otherwise stated.

### 4.2. Determination of the Antibiotic Sensitivity Spectrum of A. baumannii

The antibiotic sensitivity of *A. baumannii* was determined using the antibiotic disc diffusion method for 17 antibiotics belonging to 12 antibiotic classes with the BD BBL™ Sensi-Disc™. Briefly, the *A. baumannii* culture was spread on Muller Hinton Agar (MHA) plates, and antibiotic discs were placed on the plates aseptically and incubated at 25 °C for 24 h. Then, the diameter of the zone of inhibition was measured around the disc (6 mm). Based on the standard zone diameter interpretive chart), the antibiotic sensitivity was determined for *A. baumannii* strain, and they were identified as resistant, intermediate resistant, or susceptible.

### 4.3. Determination of the Time-Kill Kinetics, MIC, and MBC of Octominin for A. baumannii

The time-kill kinetics and MIC were determined using a microdilution susceptibility test, and MBC was determined with the agar plating method, according to the guidelines ofClinical and Laboratory Standards Institute (CLSI), M07-A. The *A. baumannii* culture (1 × 10^6^ CFU/mL) was added to a 96-well microplate (190 µL/well) in triplicate. Ten microliters of Octominin at different final concentrations (0–20 µg/mL) was added to each well. Similarly, chloramphenicol at different final concentrations (0–100 µg/mL) was used as the positive control. The plate was incubated at 25 °C for 24 h, and the bacterial growth was measured at OD_595_ at 3 h intervals (0, 3, 6, 9, 12, 18, and 21) using a microplate spectrophotometer (Bio-Rad, Saint Louis, MO, USA) for the time-kill kinetic assay. The lowest concentration that showed no OD_595_ difference (no visible growth) after 24 h was determined as the MIC. MBC was tested by plating 100 µL of bacterial suspensions from the MIC assay wells at peptide concentrations equal to or higher than the MIC values on Tryptic Soy agar (TSA) plates. The lowest concentration that produced no bacterial colonies on the plates after incubation at 25 °C for 24 h was considered as the MBC.

### 4.4. Determination of A. baumannii Cell Viability after Octominin Treatment

The MTT assay on the Octominin-treated *A. baumannii* was conducted according to the method described by Dananjaya et al. [51]. In brief, 2 mL of *A. baumannii* culture (1 × 10^6^ CFU/mL) was treated with different concentrations (0–10 µg/mL) of Octominin, and chloramphenicol (50 µg/mL) was used as the positive control. After incubation at 25 °C for 24 h, the samples were centrifuged at 1500× *g* for 10 min, and the cells were washed with PBS. The cells were incubated with 20 µL of 5 µg/mL MTT reagent (Sigma Aldrich, Munich, Germany) for 30 min. Then, dimethyl sulfoxide (DMSO) (Sigma Aldrich, Munich, Germany) was added to the samples, and the cells were resuspended. OD_595_ was measured using a microplate spectrophotometer.

### 4.5. Effect of Octominin on Morphological Changes in A. baumannii

FE-SEM analysis was conducted on the Octominin-treated *A. baumannii group* according to the method described by Jayathilaka et al. [52], with some modifications. *A. baumannii* (1 × 10^6^ CFU/mL) were treated with Octominin at the MIC and MBC levels. Chloramphenicol was used as the positive control, and PBS was used as the negative control. After incubation at 25 °C for 9 h, all samples were centrifuged at 1500× *g* for 10 min. Then, the bacterial cell pellets were washed with PBS and pre-fixed with 2.5% glutaraldehyde for 30 min. The bacterial pellets were washed with PBS and serially dehydrated with a series of different concentrations of ethanol (30%, 50%, 70%, 80%, 90%, and 100%). The cells were coated with platinum using an ion sputter (E-1030, Hitachi, Tokyo, Japan) and observed under an FE-SEM (MERLIN™, Carl Zeiss, Oberkochen, Germany).

### 4.6. Effect of Octominin on Membrane Permeability Alteration in A. baumannii

The PI uptake assay described by Jayathilaka et al. [52] was conducted, with some modifications, to detect alterations in the membrane permeability of *A. baumannii* upon treatment with Octominin. In this study, the PI uptake assay was coupled with FDA staining. In brief, the MIC (5 µg/mL) and MBC (10 µg/mL) of Octominin, 50 µg/mL of chloramphenicol (positive control), and PBS (negative control)-treated *A. baumannii* were incubated at 25 °C for 10 h. The cells were pelleted after centrifugation at 1500× *g* for 10 min and resuspended in 1 mL PBS. The bacterial cells were incubated with 50 µg/mL of PI (Sigma Aldrich, Munich, Germany) and 40 µg/mL of FDA (Sigma Aldrich, Munich, Germany) at room temperature (26 ± 2 °C) for 30 min in the dark. Excess PI and FDA were washed using PBS, and the cells were observed under a confocal laser scanning microscope (CLSM) (LSM5 Live Configuration Variotwo VRGB, Zeiss, Oberkochen, Germany). The red fluorescence was measured at excitation and emission wavelengths of 535 and 617 nm, respectively, and the green fluorescence was measured at excitation and emission wavelengths of 488 and 535 nm, respectively.

### 4.7. Effect of Octominin on ROS Generation in A. baumannii

H_2_DCFDA staining, as described by Nikapitiya et al. [22], was conducted on *A. baumannii* to detect the ROS generation following Octominin treatment. Briefly, *A. baumannii* cultures were treated with the MIC (5 µg/mL) and MBC (10 µg/mL) of Octominin, 50 µg/mL of chloramphenicol (positive control), and PBS (negative control) and incubated at 25 °C for 10 h. Then, cell pellets were obtained by centrifugation at 1500× *g* for 10 min and washing with PBS. The cells were resuspended in 1 mL of PBS and stained using H_2_DCFDA (Invitrogen, Carlsbad, CA, USA) for 30 min at room temperature in the dark. Excess H_2_DCFDA was then washed, and the cells were observed under CLSM for the detection of the green fluorescence using an excitation wavelength of 488 nm and an emission wavelength of 535 nm.

### 4.8. Effect of Octominin on LPS Neutralization

The LPS neutralization ability of Octominin was tested according to the method described by Madanchi et al. [53] using a Pierce™ LAL Chromogenic Endotoxin Quantitation Kit (Thermo Fisher Scientific, Waltham, MA, USA). First 0.50 EU/mL (0.05 ng/mL) of an LPS standard stock solution was prepared using the *Escherichia coli* endotoxin standard (available in LAL kit) and endotoxin free water (EFW). Then, 50 µL of the standard solution was placed in Eppendorf tubes and incubated at 37 °C for 5 min. The Octominin treatment (50 µL) was then conducted in a dilution series (0–100 µg/mL), and the plate was incubated at 37 °C for 30 min. After neutralization, the remaining LPS was quantified according to the manufacturer’s protocol of the LAL quantification kit. First, LAL was added to each tube, and each tube was then shaken and incubated at 37 °C for 10 min. Then, Chromogenic substrate (100 μL) was added to each tube and incubated at 37 °C for 6 min. Finally, a stop reagent was added to each tube, and 200 µL was placed from each tube into a 96-well microplate. The absorption of each sample was measured at 410 nm using a microplate spectrophotometer. The LPS neutralization percentage was quantified following Equation (1):

LPS neutralization % = (1−(Ab _test_/ Ab _negative control_)) × 100% (1)

where Ab _test_ represents the absorbance value of the Octominin-treated sample, and Ab _negative control_ represents the absorbance values of the EFW-treated sample.

### 4.9. Effect of Octominin on A. baumannii Biofilm Formation Inhibition and Eradication

To determine the effect of Octominin on *A. baumannii* biofilm formation inhibition and eradication, we conducted antibiofilm assays using crystal violet (CV) staining, as described by Kim et al. [31]. For the biofilm formation inhibition assay, *A. baumannii* (1 × 10^6^ CFU/mL) in Tryptic Soy broth (TSB), supplemented with 0.2% glucose, was added to a 96-well plate with Octominin (10 µL) at different final concentrations (0–12.5 µg/mL) and incubated at 25 °C for 24 h.

For the biofilm eradication assay, *A. baumannii* (1 × 10^6^ CFU/mL) in TSB, supplemented with 0.2% glucose, was added to a 96-well plate (100 µL/well), and the plate was incubated at 25 °C for 24 h until biofilm formation. After 24 h, the supernatant was removed, and the walls were washed carefully with PBS. Then, TSB, supplemented with 2% glucose, was replaced in each well and treated with Octominin (0–12.5 µg/mL). The plate was then incubated at 25 °C for 24 h.

The remaining biofilms in the inhibition and eradication assays were quantified using the CV method. The supernatant containing planktonic bacteria was removed, and the walls were washed with PBS. Then, 100% methanol was added to the wells to fix the biofilm, which was washed after 10 min. The biofilms were stained with 0.1% (*w*/*v*) CV (Sigma-Aldrich, Munich, Germany) for 30 min. The excess CV was removed by washing three times with PBS. Finally, the biofilm was fully dissolved in 95% ethanol, and the absorbance was measured at 595 nm using a microplate spectrophotometer. The percentage inhibition of the biofilm formation was determined using the following Equation (2):

Biofilm formation inhibition/eradication % = (1–(Ab _test_ /Ab _negative control_)) × 100% (2)

where Ab _test_ represents the absorbance value of the Octominin- or chloramphenicol-treated test group; and Ab _negative control_ represents the absorbance value of the negative control.

### 4.10. Determination of Octominin Toxicity

#### 4.10.1. Hemolysis Assay of Octominin

A hemolysis assay was conducted on mouse red blood cells (RBCs) treated with Octominin, as described by Kim et al. [31]. The RBCs were washed and suspended in PBS. Then, Octominin was added at a concentration of 0–500 µg/mL. One percent (*v*/*v*) of Triton X-100 (Sigma Aldrich, Munich, Germany) was added as the positive control. PBS was used as the negative control. After 1 h of incubation at room temperature, the supernatant was separated by centrifugation. The absorbance of the supernatant (200 µL) was measured at 415 nm using a microplate spectrophotometer (Bio-Rad, Saint Louis, MO, USA). The hemolysis percentage of RBCs was calculated using the following Equation (3) [31]:

Hemolysis % = ((Ab _test_–Ab _PBS_) / (Ab _triton_−Ab _PBS_)) × 100% (3)

where Ab _test_ represents the absorbance of the Octominin-treated RBC supernatant, Ab _pbs_ represents the absorbance of the PBS-treated RBC supernatant, and Ab _triton_ represents the absorbance of the Triton X-100-treated RBC supernatant.

#### 4.10.2. Cytotoxicity of Octominin against Murine Macrophage Cells

The MTT assay was performed for the Octominin-treated murine RAW 264.7 cells. The cells were cultured in Dulbecco’s modified Eagle’s medium (Sigma-Aldrich, Munich, Germany), containing 10% (*v*/*v*) fetal bovine serum (Sigma-Aldrich, Munich, Germany) and an antibiotic-antimycotic solution (Thermo Fisher Scientific, Waltham, MA, USA), and incubated at 37 °C in a humidified atmosphere at 5% CO_2_ for 24 h. The cells were then seeded at a density of 2.0 × 10^5^ cells/mL (100 µL/well) in a 96-well flat-bottom microtiter plate and allowed to adhere to the wells for 12 h. The culture media was replaced and treated with different concentrations of Octominin (20–500 µg/mL) and 10 µL of PBS as the negative control and incubated under the same conditions for 24 h. The culture medium was then replaced with 90 µL of fresh medium, and 10 µL of 5 µg/mL MTT (Sigma-Aldrich, Munich, Germany) was added to each well and incubated for 4 h at 37 °C. The culture medium was then removed and 50 μL of DMSO (Sigma-Aldrich, Munich, Germany) was added to solubilize the formazan dye. The absorbance was measured at 595 nm using a microplate spectrophotometer.

### 4.11. Determination of the In Vivo Efficiency of Octominin in Controlling A. baumannii Infection in a Zebrafish Model

All zebrafish experiments were conducted according to the institutional animal ethics guidelines and under the supervision of the committees of Chugnam National University. The AB wild-type zebrafish (average body weight: 400 mg) were maintained under optimal culture conditions using an automated water circulating system (14 h: 10 h, light: dark cycle, at 28 ± 0.5 °C, conductivity of 500 + 50 µS/sm, feeding equivalent to 4% of their body weight per day) throughout the experimental period. 

To determine the infective dose of *A. baumannii*, 20 µL of bacterial suspensions in PBS at different OD_595_ values (1.2, 1.3, 1.4, and 1.5) were injected intraperitoneally into the zebrafish (5 fish/group). The fish were maintained at 28 °C and monitored for mortality. Depending on the survival data, the optimum minimum infective bacteria broth was selected, and the CFU/mL was detected by plating on agar plates.

The effectiveness of Octominin in controlling *A. baumannii* infection was assessed by a simultaneous *A. baumannii* challenge and Octominin treatment. The zebrafish were divided into three groups in 5 L tanks (10 fish/ tank and two replicates/group): (1) uninfected control; (2) *A. baumannii*-infected and PBS-injected negative control group; and (3) *A. baumannii*-infected and Octominin-treated group. Initially, the zebrafish were anesthetized using water containing 160 μg/mL of buffered tricaine (ethyl 3-aminobenzoate methanesulfonate), and 20 µL of *A. baumannii* (2.1 × 10^11^ CFU/mL) was i.p. injected into the treatment group and negative control group, while the fish in the uninfected group were injected with 20 µL of PBS. The treatment was performed simultaneously. The Octominin group was injected with 10 µL of 10 mg/mL Octominin (100 µg/fish), while the other groups were injected with 10 µL of PBS. The fish were placed in the same tanks after the treatment, the tanks were maintained at 28 °C, and the fish were observed for 42 h for mortality. The pathological characteristics were monitored among the groups using light microscopy (Leica KL300 LED, Wetzlar, Germany). After 42 h, the fish were sacrificed, and the gill, spleen, and kidney were isolated to determine the bacterial burden at each organ level and for histological analysis. To determine the bacterial burden in the spleen and kidney, each organ was weighed, homogenized in 200 µL of PBS, serially diluted, and plated on TSA plates. The plates were incubated at 25 °C for 12 h, and the CFUs were counted.

Histological analysis was conducted to determine the alterations at the tissue level of zebrafish gills, kidneys, and spleens in all three groups. Briefly, the collected tissues were processed, embedded, and sectioned to a thickness of 4 µm. The sectioned samples were placed on glass slides and stained with hematoxylin and eosin (H&E), according to the method described by Liyanage et al. [54]. The slides were observed under a light microscope (Leica 3000 LED, Wetzlar, Germany).

### 4.12. Statistical Analysis

All experimental data were analyzed using the GraphPad Prism software version 5 (GraphPad Software Inc., La Jolla, CA, USA). One-way analysis of variance (ANOVA) and/or unpaired t-test were performed to determine the statistically significant (*p* < 0.05) differences between the control and the treatments of MTT, hemolysis, biofilm inhibition, biofilm eradication assays, and bacterial burden results. The in vivo survival data were analyzed using a Log-rank (Mantel-Cox) test to identify the significance (*p* < 0.05) levels for the *A. baumannii*-infected negative control (PBS) vs *A. baumannii*-infected and Octominin-treated groups. The data are shown as the mean ± standard deviation (SD) of triplicate experiments.

## 5. Conclusions

*A. baumannii* (Lab strain 1) showed MDR against the conventional antibiotics tested. Octominin was capable of suppressing the growth of *A. baumannii* isolates at a low MIC of 5 µg/mL, and its bactericidal activity was at a relatively low MBC (10 µg/mL). Octominin exerts its antibacterial activity against *A. baumannii* with multiple modes of action, such as morphological and structural alterations and damage to the bacterial surface, induction of membrane permeability, induction of ROS generation, LPS neutralization, inhibition of biofilm formation, and biofilm eradication. In addition, Octominin showed a very low cytotoxicity in mammalian cells at higher concentrations (>100 µg/mL). Furthermore, an in vivo zebrafish study confirmed the efficient control of *A. baumannii* with Octominin, with a low mortality rate and minimal damage to the fish at the tissue level. Considering all these key points, Octominin is a promising lead molecule to be developed as a novel and effective AMP for therapeutic use to overcome the complications of MDR *A. baumannii* infections.

## Figures and Tables

**Table 1:** Antibiotic sensitivity spectrum of *A. baumannii* (Lab strain 1).

Antibiotic Class	Antibiotic	Diameter of Zone of Inhibition (mm)	Antibiotic Sensitivity Level
Aminoglycoside	Streptomycin (300 µg)	13	Resistant
	Gentamycin (120 µg)	14	Resistant
	Amikacin (30 µg)	15	Intermediate
Tetracyclines	Tetracycline (30 µg)	16	Intermediate
	Doxycycline (30 µg)	20	Susceptible
Fluoroquinolone	Ciprofloxacin (5 µg)	19	Intermediate
	Ofloxacin (5 µg)	17	Susceptible
Glycopeptide	Vancomycin (30 µg)	0	Resistant
Macrolides	Erythromycin (15 µg)	0	Resistant
	Tobramycin (10 µg)	11	Resistant
Cephalosporin	Cefotaxime (30 µg)	10	Resistant
Penicillin	Penicillin (10 U)	0	Resistant
Lincosamide	Clindamycin (2 µg)	0	Resistant
Carbapenem	Imipenem (10 µg)	25	Susceptible
Folic acid synthesis inhibitor	Sulfamethoxazole/ Trimethoprim (23.75/1.25 µg)	15	Intermediate
Diaminopyrimidine	Trimethoprim (5 µg)	0	Resistant
Rifamycin	Rifampin (5 µg)	0	Resistant
Chloramphenicol	Chloramphenicol (30 µg)	22	Susceptible

## Data Availability

The data presented in this study are available on request from the corresponding author.
